# NEK2 regulates cellular proliferation and cabergoline sensitivity in pituitary adenomas

**DOI:** 10.7150/jca.52937

**Published:** 2021-02-05

**Authors:** Fangfang Jian, Yuhao Sun, Qingfang Sun, Benyan Zhang, Liuguan Bian

**Affiliations:** 1 Department of Obstetrics and Gynecology, Ruijin Hospital, Shanghai Jiao Tong University School of Medicine, Shanghai, China.; 2 Department of Neurosurgery, Ruijin Hospital, Shanghai Jiao Tong University School of Medicine, Shanghai, China.; 3 Department of Pathology, Ruijin Hospital, Shanghai Jiao Tong University School of Medicine, Shanghai, China.

**Keywords:** prolactinoma, NEK2, USP7, cabergoline sensitivity

## Abstract

**Objective:** To identify critical roles played by NEK2 in prolactinomas and to clarify the corresponding underlying mechanisms.

**Methods:** We performed RNA-seq on MMQ cell lines treated with the dopamine receptor agonist cabergoline (CAB) to identify genes involved in prolactinoma progression and dopamine receptor-agonist (DA) sensitivity. NEK2 was then selected for further study. The expression of NEK2 was examined using quantitative real-time PCR, western immunoblotting, and immunohistochemistry - both in pituitary adenomas (PA) and in normal pituitary tissue. We used gain-of-function and loss-of-function assays to explore the biologic roles of NEK2 in cell growth *in vivo* and *in vitro*. Co-immunoprecipitation was also used to detect the binding between NEK2 and USP7.

**Results:** Herein, we reported that NEK2 was upregulated in prolactinomas, particularly dopamine-resistant prolactinomas. NEK2 overexpression significantly promoted pituitary tumor GH3 and MMQ cell proliferation, and it impaired cellular sensitivity to CAB. Conversely, knockdown of NEK2 inhibited GH3 and MMQ cell growth, and sensitized the cells to CAB. Mechanistically, NEK2 regulated cell proliferation via the Wnt-signaling pathway; and in addition, we demonstrated that USP7 interacted with, deubiquitylated, and stabilized NEK2.

**Conclusions:** Collectively, our results suggest that NEK2 might be a potential therapeutic target for prolactinoma.

## Introduction

Pituitary adenomas (PA) are principally benign intracranial tumors; and prolactinomas constitute the most frequently observed functional pituitary tumors - representing nearly 40% of all adenomas of the pituitary gland, with a prevalence of 100 per million population 1, 2. Clinically, dopamine receptor agonists (DAs) (primarily bromocriptine [BRC] and cabergoline [CAB]) are the first-line treatment for the majority of patients with prolactinomas, and they effectively suppress prolactin secretion and shrink tumor volume in most patients 3. However, tumor resistance is approximately 20%-30% and 10% to BRC and CAB, respectively 3. Therefore, discovering markers that can identify the proliferative character of prolactinomas and DA sensitivity is crucial to developing novel therapeutic strategies.

Never in mitosis (NIMA)-related kinase 2 (NEK2) is a serine/threonine kinase that promotes centrosome splitting and ensures correct chromosomal segregation during the G2/M phase of the cell cycle 4. Recently accumulating evidence has revealed that NEK2 overexpression occurred in several neoplastic diseases - including hepatocellular carcinoma 5, colorectal cancer 6, lung cancer 7, prostate cancer 8, breast cancer 9, and glioma 10. Although these studies also suggested that NEK2 overexpression conferred reduced survival with various tumors, the biologic role played by NEK2 in prolactinoma remains to be elucidated.

To identify genes involved in the progression of PRL-secreting pituitary adenomas and DA sensitivity, we executed RNA-seq on MMQ cell lines treated with the dopamine receptor agonist cabergoline. A total of 589 genes were identified that were induced or repressed; and of these, NEK2 was significantly diminished after CAB treatment. Furthermore, we reported that NEK2 was upregulated in prolactinoma tissues, especially dopamine-resistant prolactinomas. We observed that the inhibition of NEK2 inhibited PA cell growth *in vitro* and *in vivo*, and enhanced drug-induced responses. We also determined the effects of NEK2 overexpression on cell growth and drug resistance, and explored the regulation of NEK2 stability. Collectively, these data suggest that NEK2 occupies an important role in prolactinoma progression, and is thus a potentially novel therapeutic strategy for prolactinomas.

## Materials and Methods

### Cell culture and other reagents

Rat prolactinoma GH3 and MMQ cell lines were purchased from the American Type Culture Collection and cultured in Dulbecco's modified Eagle's medium (DMEM, 11965, GibcoTM) and F12 medium, respectively - supplemented with 2.5% fetal bovine serum and 15% horse serum (04-124-1A, Biological Industries), 100 U/mL penicillin, and 100 μg/mL streptomycin (15140122, GibcoTM). All reagents were purchased from Sigma Aldrich unless stated otherwise.

### Pituitary tumor samples

Thirty-six human prolactinoma tissue samples (including twenty-six DA-sensitive and ten DA-resistant) were obtained from pituitary tumour patients diagnosed as prolactinoma who underwent surgery between 2013 and 2018 at the Department of Neurosurgery, Ruijin Hospital affiliated to Shanghai Jiaotong University. Pharmacologic resistance to dopamine agonists is defined here as failure to normalize PRL levels and failure to decrease macroprolactinoma size by ≥50% 11. The clinical information and laboratory test results for these 36 prolactinomas are listed in Supplementary Table 1. Seven normal human pituitary gland specimens were obtained from autopsy. The procedures related to human subjects were approved by the Ethics Committee of Shanghai Jiao Tong University School of Medicine.

### Cell proliferation assays and colony formation assay

Cells were seeded in 96-well plates at a density of 5000 cells in quintuplicate. After 24, 48, 72, and 96 h, the number of viable cells was measured with a CellTiter-Glo luminescence cell-viability assay (G3588, Promega). Upon addition of MTS solution, the plate was incubated at 37 °C for 2 h, and we determined absorbance at 490 nm with a plate reader (Tecan, Switzerland).

To assess colony formation, we plated cells at a density of 1000 cells/well in a 6-well plate. After 2 weeks, cells were fixed with 4% paraformaldehyde and stained with 0.1% crystal violet solution for 20 min at room temperature. The plates were photographed after extensive washings and air drying.

### 5-Ethynyl-2'-deoxyuridine staining

We performed 5-ethynyl-2'-deoxyuridine** (**EdU) staining using a Click-iT EdU Alexa Fluor 647 Imaging Kit (Invitrogen) according to the manufacturer's protocol. Cells were incubated with 10 μM EdU for 12 h, and then fixed and permeabilized for EdU detection. We then analyzed the percentage of EdU-positive cells.

### Immunohistochemistry

Tissue samples were fixed in 4% formalin and embedded in paraffin. Tissue slices were cut into 5 μm thick. 7 normal pituitary tissues and 36 prolactinoma specimens were used. A total of 7 normal and 32 tumor samples had sufficient tissue for unambiguous analyses. Paraffin sections were dewaxed with xylene and rinsed with ethanol. Antigens were retrieved in EDTA buffer by boiling. Sections were stained with primary antibodies (NEK2 antibody, sc-55601, santa cruz) overnight at 4 °C and horseradish peroxidase-conjugated secondary antibodies (Dako) for 30 min at room temperature. For histological analysis, dewaxed sections were counterstained with hematoxylin and eosin. Clinicopathological characteristics of stained slices were assessed by two pathologists blinded to the patients' clinical features. All tissues were assigned a score based on staining intensity (0, no staining; 1, low positive; 2, positive; 3, high positive).

### Western immunoblotting and immunoprecipitation

Total cell lysate was prepared with RIPA buffer containing a Protease Inhibitor Cocktail, and protein concentrations were measured with a BCA protein assay kit (Pierce). Proteins were loaded equally on 10% SDS-polyacrylamide gels, transferred to polyvinylidene difluoride membranes by electrophoresis, incubated with primary and secondary antibodies, and finally visualized using a chemiluminescence detection kit (Millipore). The primary antibodies we used were as follows: non-phospho (active) β-catenin (8814, Cell Signaling Technology [CST]), caspase-3 (9662, CST), cleaved caspase-3 (9664, CST), USP7 (4833, CST), NEK2 (ab115731, abcam), cyclin D1 (55506, CST), c-myc (18583, CST), phosphor-erk1/2 (4370, CST), Erk1/2 (4695, CST), GAPDH (2118, CST), α-tubulin (2125, CST), GFP (2956, CST), and ubiquitin (3936, CST).

For immunoprecipitation (IP) assays, cells were lysed in lysis buffer for 20 min. Lysates were then cleared by centrifugation and filtered through 0.45-μm spin filters (Millipore) to further remove cellular debris, and the resulting material was subjected to IP with 50 μL of anti-FLAG M2 affinity resin (Sigma) overnight at 4 °C. Resin-containing immune complexes were washed with ice-cold lysis buffer followed by Tris-buffered saline (TBS) washes. Proteins were eluted with 2-50-μL aliquots of 150 μg/mL 3× Flag-peptide (Sigma) in TBS for 30 min, and the elutions subsequently pooled for a final volume of 100 μL. Proteins in each elution were ultimately precipitated with cold acetone.

### Quantitative real-time PCR

Total RNA was extracted with TRIzol reagent (Invitrogen, Carlsbad, CA). For reverse transcription, 1 μg of the total RNA was converted to cDNA in a 20-μL reaction volume using a reverse-transcription kit (Promega) following the manufacturer's instructions. Real-time PCR was carried out on the LightCycler 480 system (Roche) using SYBR Green Supermix (Takara). We calculated relative RNA levels using the 2^-ΔCT^ method and normalized them to β-actin or GAPDH mRNA levels. Quantification was performed in quadruplicate, and the experiments were repeated independently three times. The primer sequences are shown in Supplementary Table 2.

### Cell apoptosis assay

Analysis for cellular apoptosis was performed with the Annexin V-FITC/PI Apoptosis Assay Kit (BD Biosciences, USA) according to the manufacturer's instructions, and we detected apoptotic rates by flow cytometry (BD FACSCanto II, USA).

### Luciferase assays

For the luciferase reporter assays, GH3 and MMQ cells were seeded in 24-well plates and co-transfected with 1 μg of TOP (T-cell factor reporter plasmid)- or FOP (mutant T-cell factor reporter plasmid)-flash luciferase, using Lipofectamine 3000 (Invitrogen). For each transfection, 0.4 μg of β-galactosidase cDNA was co-transfected to normalize transfection efficiency. After 48 h, the cells were lysed with luciferase lysis buffer for 20 min; and luciferase activity was measured using the Dual Luciferase Reporter Assay System (Promega).

### GH3 xenograft mouse model

Six-week-old nu/nu male mice were inoculated subcutaneously with GH3 cells (6×10^5^ cells per mouse) suspended in 100 μL of PBS into 1 flank of nude mice. Each group comprised 5 mice, and tumor size was measured by caliper measurements every 2 days. We calculated tumor volume using the formula 1/2 × (length × width^2^). Thirty days after cell inoculations (each group was composed of 8 mice), animals were euthanized, tumors excised and weighed, and blood samples were collected. The animal protocol was reviewed and approved by the Animal Care Committee of Shanghai Jiao Tong University School of Medicine.

### Statistics

We performed statistical analyses using SPSS version 16.0. Values are described as means ± standard deviation. Significant differences were analyzed using a two-tailed, unpaired Student's *t*-test, 1-way and 2-way ANOVA. The Mann-Whitney U-test was used for continuous variables. A *P* value less than 0.05 was considered statistically significant.

## Results

### NEK2 is upregulated in human pituitary tumors

**Figure 1:**
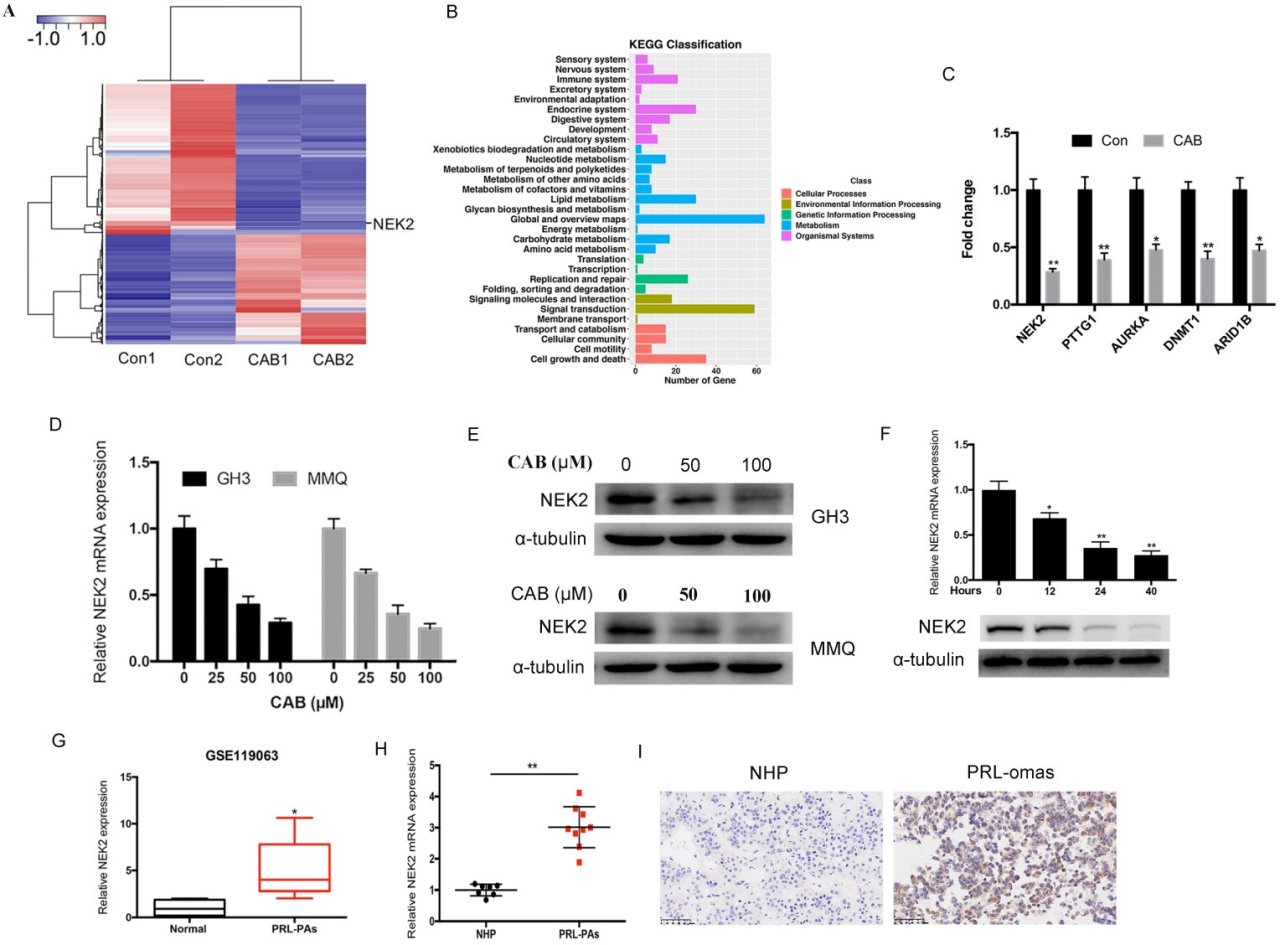
NEK2 expression is increased in PRL-omas. (A) Heatmap of significant differentially expressed mRNAs between controls and CAB-treated MMQ cells. (B) KEGG pathway analysis. (C) mRNA expression of NEK2, PTTG1, AURKA, DNMT1, and ARID1B was decreased after CAB treatment. D and E, GH3 and MMQ cells were treated with different doses of CAB. Immunoblotting and qPCR analyses of NEK2. (F) CAB inhibited NEK2 expression in a time-dependent manner. (G) NEK2 expression was increased in a human prolactinoma (GEO dataset GSE119063). (H) NEK2 mRNA expression was increased in human prolactinomas (n=9) compared with normal pituitary glands (n=7). (I) Immunohistochemical (IHC) staining of NEK2 in normal pituitary and PRL-secreting pituitary adenomas. Thirty-two specimens were immunohistochemically stained with indicated antibodies. Representative photos of tumors are shown.

We first evaluated the half-inhibitory concentration (IC50) of the dopamine agonist cabergoline (CAB) in PA cell lines. MMQ cells were incubated with various doses of CAB ranging from 5 to 200 μM for 24 and 48 h. Then, the IC50 values were calculated by cell viability assay. The IC50 of MMQ cells was approximately 48.2 μM at 24 h and 41.9 μM at 48 h (Supplementary Figure 1), which was consistent with previous research 12. Then RNA-seq was performed on MMQ cell lines that were stimulated with 50 μM CAB for 24 h. A total of 589 mRNAs were identified, and of these, 336 mRNA were down-regulated and 253 mRNA were up-regulated (Figure 1A). KEGG pathway analysis showed that the major signaling pathways for these genes were signal transduction, cell growth and death, and replication and repair (Figure 1B) - suggesting that these genes are related to DA resistance in prolactinomas. Among the differentially expressed genes, PTTG1, E2F1, Mki67, CCNB1, and CCNA2 were significantly reduced after CAB treatment - demonstrating the reliability of our sequencing results. We were particularly interested in NEK2, AURKA, ARID1B, PLK1, MDM1, DNMT1, CDC25C, and BRAC1, as they are involved not only in chromosomal instability, but also in cancer cell proliferation and drug resistance. We focused on NEK2, because it was most markedly downregulated by approximately 3.5-fold after CAB treatment. Quantitative real-time PCR (qPCR) analysis also showed that NEK2, PTTG1, AURKA, DNMT1, and ARIDIB were significantly decreased by CAB treatment (Figure 1C). To further validate NEK2 gene regulation by DAs, we determined NEK2 mRNA and protein levels following CAB treatment, and found that NEK2 expression was inhibited in GH3 and MMQ cells by CAB in a dose- and time-dependent manner (Figure 1D-F). Moreover, analysis of previous microarray datasets from the gene expression Omnibus repository database showed that NEK2 expression was augmented in prolactin tumors compared to normal pituitary tissues (GSE119063) (Figure G). We then assessed the expression of NEK2 in our PA tissues and normal human pituitary glands by immunohistochemistry (IHC) and qPCR, which also corroborated the upregulation of NEK2 in PRL-omas (Figure 1H and I). These data suggested that NEK2 may be associated with PA progression.

### NEK2 promotes tumor cell proliferation both *in vitro* and *in vivo*

**Figure 2:**
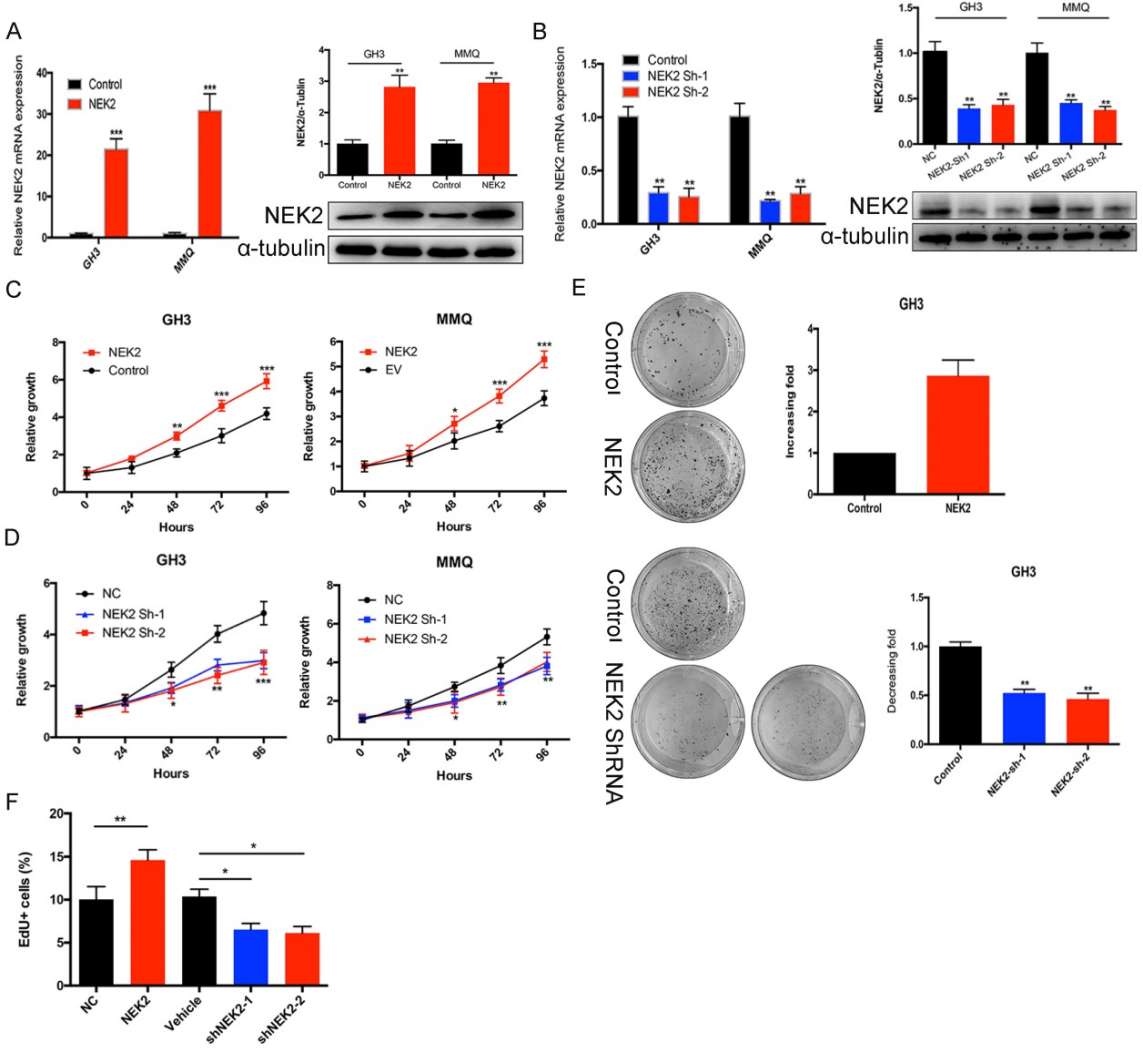
NEK2 promotes the proliferation of PA cells *in vitro*. (A and B) The efficiency of NEK2 overexpression and knockdown by qPCR and WB. (C and D) MTS assays were performed to evaluate the proliferation of transfected cells each day for 4 days. NEK2 overexpression promoted GH3 and MMQ cell proliferation, while NEK2 knockdown inhibited proliferation. (E) Colony formation assays performed on GH3 cells infected with lentivirus overexpressing NEK2 or shRNA construct. (F) Percentage of EdU-positive cells was analyzed in the EdU (at 10 µM for 12 h)-treated cells 36 h after transfection.

**Figure 3:**
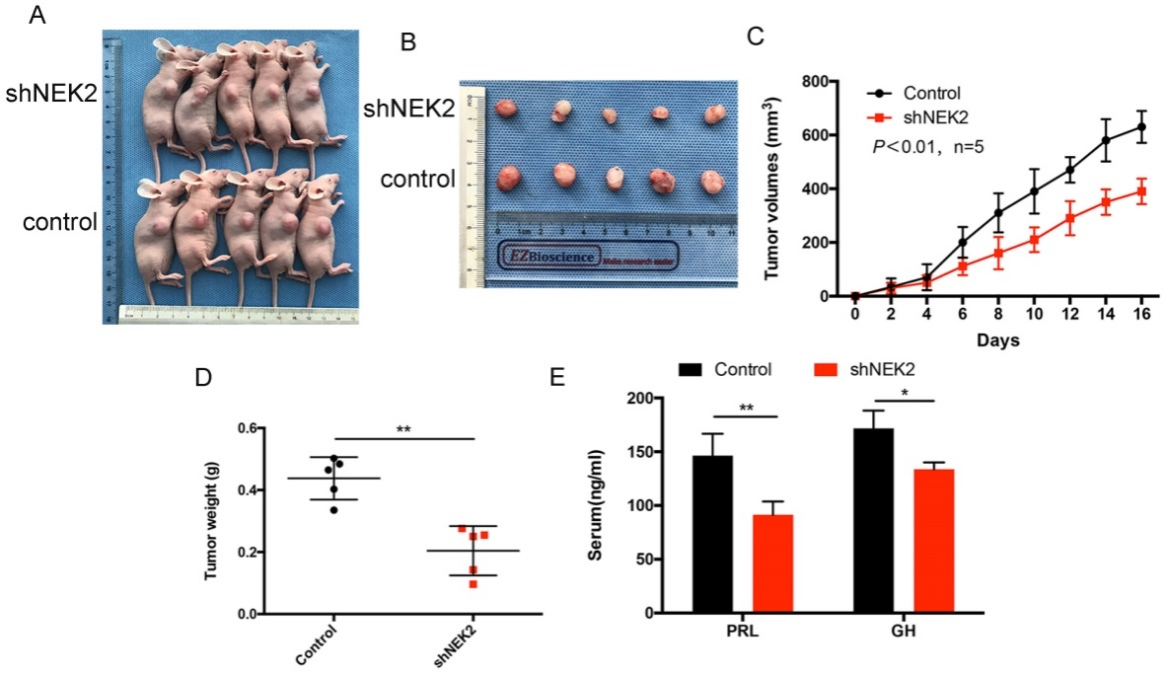
NEK2-knockdown inhibits GH3 tumor growth *in vivo*. (A and B) Images of nude mice and tumors. GH3 cells manifesting NEK2 knockdown or vehicle treatment were subcutaneously grafted into nude mice, and tumor volumes were calculated after injection every 2 days. At the end of the experiments, mice bearing tumors were sacrificed, and the xenograft tumors were dissected and weighed (D). (C) *In vitro* growth curves of the pituitary adenoma cell line GH3 expressing vehicle or shNEK2. (E) Serum prolactin and GH on day 16 (ng/mL) after NEK2 knockdown.

To investigate the role of NEK2 in prolactinomas, we stably overexpressed and knocked down NEK2 by lentiviral mediation in pituitary tumor GH3 and MMQ cells, and executed cell viability and proliferation assays. Overexpression and knockdown efficiencies were evaluated using qPCR and immunoblotting analyses (Figure 2A, B). NEK2 overexpression significantly increased cell viability and colony formation compared with empty vector-transfected cells (Figure 2C, E). In addition, the 5-ethynyl-2'-deoxyuridine (EdU) proliferation assays indicated that cellular replication was significantly promoted upon NEK2 overexpression (Figure 2F). Conversely, NEK2 knockdown with 2 independent shRNAs resulted in attenuated cell proliferation and colony formation (Figure 2D, E and F). The effects of NEK2 dysregulation on tumorigenicity were then further evaluated *in vivo*. GH3 cells stably transfected with NEK2 shRNA or vehicle were inoculated in 6-week-old nude female mice; and relative to vehicle-treated control mice, NEK2 knockdown significantly inhibited tumor growth (Figure 3A, B and C). Tumors derived from shNEK2-transfected prolactin tumor cells also weighed significantly less than those from the control group (0.204±0.071 *vs* 0.437±0.061g; *P* < 0.01) (Figure 3D). Consistent with the faster tumor-growth rate and development of larger tumors in mice inoculated with vehicle-transfected cells, plasma PRL and serum GH levels were also reduced by 38% and 22%, respectively, in the shNEK2-transfected group (Figure 3E).

### NEK2 does not affect apoptosis of pituitary tumor cells

**Figure 4:**
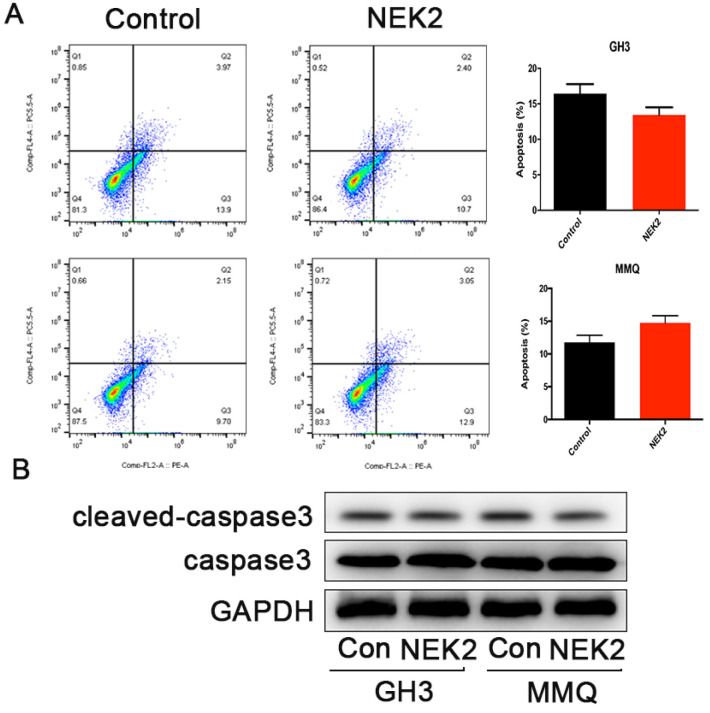
NEK2 does not affect apoptosis of pituitary adenoma cells. (A) Percentage of annexin V/PI-positive cells of GH3 and MMQ cells overexpressing NEK2 as analyzed by flow cytometry (n=3 for each group). (B) Protein levels of cleaved caspase-3 in GH3 and MMQ cells overexpressing NEK2.

To further investigate whether NEK2 inhibited cellular apoptosis, we adopted annexin V/PI staining and cleaved caspase-3 assays, and analyzed apoptotic and necrotic cell death using annexin staining by flow cytometry. The percentage of apoptotic cells in the NEK2-overexpressing cells was indistinguishable from the control cells (Figure 4A), and activated caspase-3 protein levels remained unchanged upon NEK2 overexpression (Figure 4B). Overall, these data indicated that NEK2 did not affect cellular apoptosis.

### NEK2 inhibits CAB sensitivity

**Figure 5:**
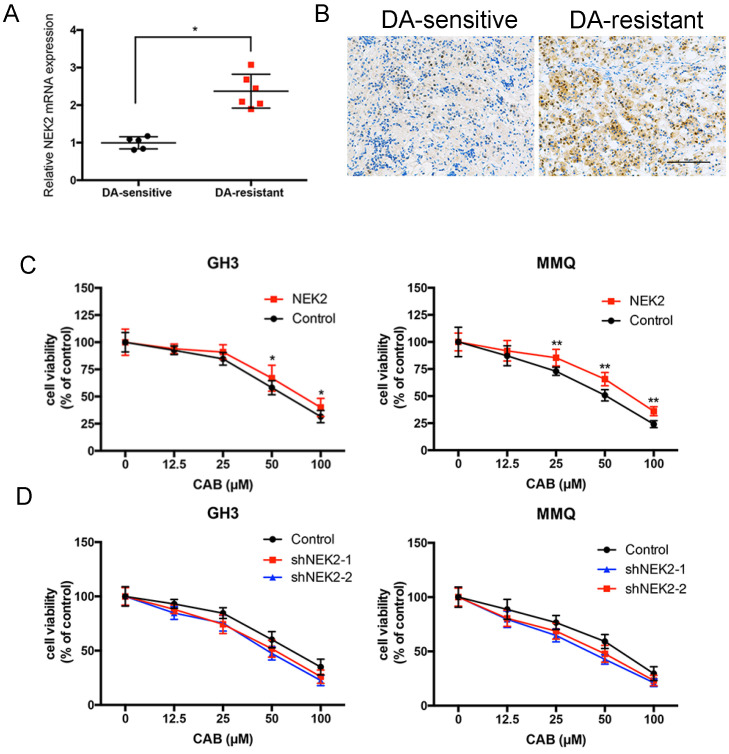
NEK2 modulates CAB sensitivity. (A and B) Both q-PCR and immunohistochemical analyses showed that NEK2 was upregulated in dopamine-resistant prolactinomas relative to sensitive tumors. (C and D) Cell viability after NEK2 overexpression or knockdown and controls as examined using a drug- sensitivity assay. Viability of cells was monitored for 48 h using a cell proliferation assay after cells were treated with various doses of CAB (**P < 0.001, *P < 0.05).

Although we previously demonstrated that NEK2 promoted tumorigenicity, whether NEK2 affects the response to dopamine agonists remains unknown. We found that NEK2 mRNA expression in 7 BRC-resistant prolactinomas was increased 2.3-fold compared to that in 11 BRC-sensitive tumors (Figure 5A). Immunohistochemical staining also consistently revealed that NEK2 expression increased in BRC-resistant prolactinomas compared with BRC-sensitive tumors (Figure 5B). To examine the effect of NEK2 on drug resistance we overexpressed and knocked down NEK2 in MMQ and GH3 cell lines and treated the cells with different doses of CAB. As shown in Figure 5A and B, overexpression of NEK2 decreased the anti-proliferative activity of CAB treatment in these cells; while, in contrast, knockdown of NEK2 was found to enhance the anti-proliferative activity of CAB treatment in both cell lines (Figure 5C and D). These data indicated that NEK2 was able to decrease DA sensitivity in prolactinoma cells.

### USP7 interacts with and deubiquitinates NEK2

**Figure 6:**
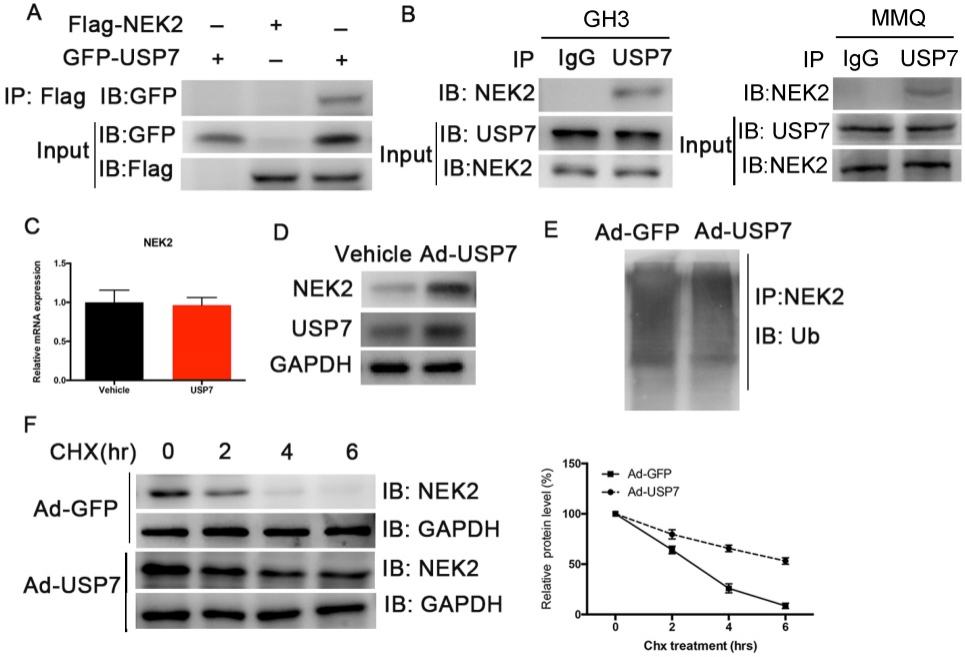
USP7 regulates the stability of NEK2. (A) NEK2 levels were assessed by immunoblotting (IB) following transfection of USP7 in 293T cells. (B) NEK2 was immuno-precipitated from GH3 and MMQ cells using anti-NEK2 or IgG antibody. (C) Relative mRNA levels of NEK2 in GH3 cells overexpressing USP7. (D) Endogenous expression of NEK2 protein was determined in GH3 cells overexpressing USP7 for 48 h. (E) NEK2 ubiquitination in GH3 cells overexpressing USP7 or GFP. Cells were pretreated with MG132 for 4 h. (F) Left: GH3 cells with or without overexpression of USP7 were treated with CHX (10 µg/mL) for the indicated times. The half-life of NEK2 was measured. Right: densitometric analysis performed on corresponding IBs to assess NEK2 half-life under the indicated conditions.

Although NEK2 expression is upregulated in various cancers, the regulation of its expression remains unclear. Studies have shown that USP7 deubiquitinates NEK2 and affects its stability 13. We therefore examined whether USP7 deubiquitinated NEK2 and affected its function in PAs, and confirmed a specific interaction between USP7 and NEK2 in transiently transfected HEK293T cells using co-immunoprecipitation (Figure 6A). The endogenous interaction of these 2 proteins was also detected in the GH3 and MMQ PA cells (Figure 6B). Because USP7 can interact with NEK2, we determined whether USP7 regulated NEK2 stability or decelerated its protein degradation. Indeed, endogenous NEK2 protein contents were dramatically increased in GH3 cells transfected with adenovirus expressing USP7, whereas its mRNA levels remained unchanged (Figure 6C and 6D). Next we examined whether the increase in NEK2 protein levels was dependent upon the deubiquitinating enzymatic activity of USP7, and found that ectopic expression of USP7 decreased NEK2 ubiquitination (Figure 6E). Furthermore, overexpression of USP7 reduced the half-life of NEK2 (Figure 6F), supporting the concept that USP7 enhances NEK2 stability. Collectively, these data suggested that NEK2 is a specific substrate of USP7 in pituitary tumor cells.

### NEK2 activates the Wnt-signaling pathway

**Figure 7:**
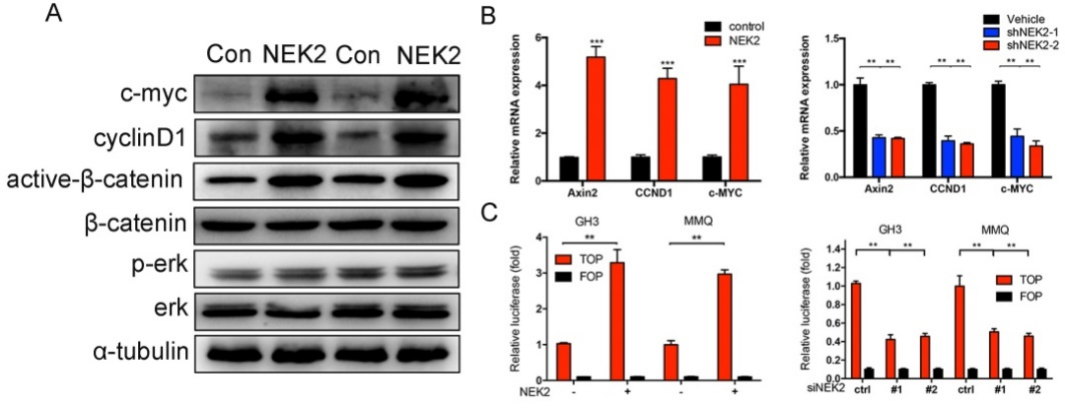
NEK2 activates the Wnt-signaling pathway in PRL-secreting pituitary tumor cells. (A) Immunoblot analysis of the key Wnt pathway-related proteins in GH3 cells transfected with NEK2 or empty vector. (B) RT-qPCR analysis of Wnt/b-catenin pathway downstream target genes in GH3 cells infected with lentivirus overexpressing a NEK2 or shRNA construct *P < 0.05, **P < 0.001, ***P < 0.0001. (C) TOP-Flash (TCF optimal promoter) assay used when NEK2 was overexpressed or knocked down in GH3 and MMQ cells.

We further explored the mechanism(s) underlying NEK2 mediation of tumor growth. Western blotting analysis revealed that overexpression of NEK2 upregulated the levels of the Wnt effectors cyclin D1, C-myc, and active b-catenin; whereas NEK2 knockdown diminished the expression of these key markers (Figure 7A). Since Wnt signaling contributes to oncogenic potential, this makes it an attractive therapeutic target that is currently being explored for cancer therapy. We therefore performed qPCR and validated Wnt-associated genes such as AXIN2, c-MYC, CCND1, and CTNNB1 as being significantly upregulated in GH3 and MMQ cells following NEK2 transfection (Figure 7B). We next evaluated whether the oncogenic properties of NEK2 were due to the activation of Wnt signaling. As expected, NEK2 overexpression in GH3 and MMQ cells enhanced the activity of the Wnt signal-transduction pathway as demonstrated by the TOPflash luciferase reporter assay; whereas knockdown of NEK2 in GH3 and MMQ cells dramatically abolished TOP-flash activity (Figure 7C). Our data thereby indicated that NEK2 activates the Wnt/β-catenin signaling pathway and plays an important role in cell growth and sensitivity to DAs.

## Discussion

In the treatment of pituitary tumors, DAs are primarily used to treat prolactinomas; and are effective in inhibiting prolactin hypersecretion, reducing tumor size, and restoring gonadal function. However, the emergence of resistance to long-term endocrine treatment is inevitable in a proportion of patients. Primary or secondary resistance to DAs thus represents challenging clinical scenarios, and this is particularly true for aggressive prolactinomas in which surgery and radiotherapy may not achieve tumor control. In such settings it is imperative to generate novel strategies to target this disease, and some investigators have thus delineated the miRNA expression profiles of dopamine agonist-resistant pituitary adenomas. Wu used Solexa sequencing to compare miRNA expression profiles between bromocriptine-sensitive and -resistant prolactinomas, and identified 12 miRNAs that were differentially expressed in bromocriptine-resistant PRL-omas 14. Xiao similarly screened 14 miRNAs that were differentially expressed in drug-resistant prolactinomas using high-throughput sequencing 15. However, the gene expression profile regulated by DAs has not yet been systematically analyzed for prolactinomas. In the current study, we therefore explored genes involved in prolactinoma progression and DA sensitivity using RNA-seq on MMQ cell lines treated for 24 h with the DA agonist cabergoline. Although our results showed that 336 mRNAs were down-regulated and 253 mRNAs were up-regulated by CAB treatment, they indicated that a large proportion of the DA-regulated transcriptome remains uncharacterized. Furthermore, KEGG pathway analysis revealed that multiple DA-regulated genes were involved in cellular growth and the secretion of prolactin - including signal transduction, cell growth and death, replication and repair, lipid metabolism, and endocrine system.

Our Seq profiling of DA-regulated genes identified the top-ranking candidate as NEK2, which we functionally characterized. Although previous studies indicated that NEK2 promoted tumor cell proliferation, tumor progression, and drug resistance 16, functional studies linking NEK2 with pituitary tumorigenesis are exiguous. Herein, we demonstrated that NEK2 promoted pituitary tumor cell growth *in vivo* and *in vitro*, and that it affected DA sensitivity. Our results also revealed a previously unappreciated pro-oncogenic role of NEK2 in prolactinomas.

Ubiquitination (which is a reversible and significant post-translational modification responsible for regulating the stability and activity of modified proteins) is involved in the regulation of nearly all biologic processes, and is associated with tumorigenesis and development 17. A plethora of key proteins implicated in oncogenesis - such as p53, PTEN, and c-Myc - have recently been revealed to be exquisitely regulated by 1 or more deubiquitinating enzymes 18, 19; and in our study we identified USP7 as a critical deubiquitinase that stabilizes NEK2.

NEK2 is associated with drug resistance in multiple cancers 13, and several publications have shown regulation of NEK2 by antitumor drugs. Deng's research demonstrated that NEK2 binds β-catenin, blocking the interaction between NEK2 and the destruction complex; ultimately contributing to sorafenib resistance in hepatocellular carcinoma 20. In Wen's study, overexpression of NEK2 in cancer cells resulted in enhanced chromosomal instability, cellular proliferation, and drug resistance; while NEK2 knockdown overcame cancer cell resistance to drugs and induced apoptosis *in vitro* and in a xenograft myeloma mouse model 21. In the present study we furthermore found that NEK2 expression was inhibited by CAB in a dose- and time-dependent manner, and that silencing of NEK2 sensitized pituitary tumor cells to CAB.

## Conclusions

In summary, our results revealed an oncogenic role for NEK2 in prolactinomas. NEK2 was upregulated in PAs and inhibition of NEK2 impaired the proliferation of pituitary tumor cell lines *in vitro* and *in vivo*. Moreover, NEK2 knockdown sensitized pituitary tumor cell lines to CAB, increasing its anti-proliferative actions by inactivating the Wnt-signaling pathway. We posit that targeting USP7 or NEK2 might have therapeutic potential in pituitary tumors, especially dopamine-resistant tumors marked by high NEK2 expression.

## Supplementary Material

Supplementary figures and tables.

## Abbreviations

BRC: bromocriptine
CAB: cabergoline
DAs: dopamine receptor agonists
NEK2: never in mitosis-related kinase 2
USP7: ubiquitin-specific peptidase 7
PRL: prolactin
PA: pituitary adenomas
qPCR: quantitative real time-PCR
TBS: Tris-buffered saline
TOP: T-cell factor reporter plasmid
FOP: mutant T-cell factor reporter plasmid
