# Supplementary material for: NEK2 regulates cellular proliferation and cabergoline sensitivity in pituitary adenomas
Source: J Cancer. 2021 Feb 5;12(7):2083–91. doi: 10.7150/jca.52937 (PMC7974539; doi:10.7150/jca.52937)
Supplement: Supplementary file 1 — Supplementary figures and tables. [file jcav12p2083s1.pdf]

Supplementary information for "NEK2 regulates cellular proliferation and cabergoline sensitivity in pituitary adenomas"

Supplementary figures

Supplementary Figure 1. MMQ cells were treated with difference doses of CAB for 24 and 48 hours. MTS assays were performed to evaluate the cell viability.

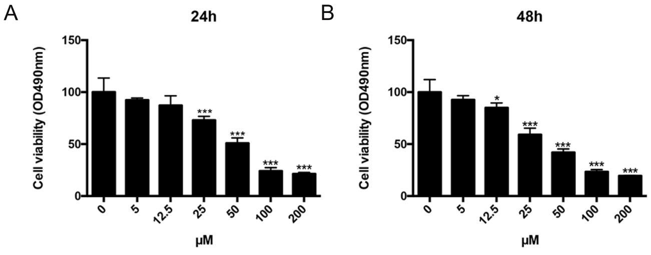

Supplementary Tables:

Supplementary Table 1.

Subtype	Gender	Ages	clinical presentation	Tumor size (mm)	Invasiveness	BRC dose (mg)	Time with BRC (Month)	immunostaining score
Sensitive								
1	F	30	amenorrhea	24*15*35	No	5	12	1+
2	F	33	lactation	15*7*16	No	2.5	36	/
3	M	37	visual loss	36*16*13	invasive	5	10	1+
4	F	34	lactation	15*10*23	No	5	15	1+
5	F	18	amenorrhea	16*16*22	No	2.5	24	2+
6	M	37	visual loss	18*25*15	No	2.5	18	1+
7	M	33	sexual dysfunction	18*17*29	No	2.5	36	1+
8	M	43	visual loss	35*17*25	invasive	5	4	0+
9	F	24	amenorrhea	13*15*29	No	1.25	36	1+
10	F	18	amenorrhea	15*27*17	No	2.5	26	1+
11	F	19	amenorrhea	27*15*23	No	2.5	24	0+
12	F	23	amenorrhea	12*17*16	No	1.25	40	/

13	M	53	headache	18*29*17	invasive	2.5	14	1+
14	F	26	amenorrhea	28*21*26	No	2.5	33	1+
15	F	32	amenorrhea	22*31*43	No	2.5	36	0+
16	F	54	visual loss	33*22*27	invasive	5	18	1+
17	M	40	headache	25*18*15	No	5	8	1+
18	M	35	sexual dysfunction	12*27*13	No	1.25	40	1+
19	M	39	headache	25*20*21	invasive	2.5	12	2+
20	F	20	lactation	17*13*18	No	1.25	48	/
21	M	44	headache	23*11*28	No	2.5	30	0+
22	F	32	amenorrhea	31*33*23	No	2.5	24	1+
23	F	21	visual loss	44*25*19	invasive	5	3	1+
24	F	24	amenorrhea	16*16*25	No	2.5	18	1+
25	F	18	amenorrhea	18*36*23	invasive	5	8	2+
26	M	34	sexual dysfunction	19*18*19	No	1.25	36	1+
Resistant								
1	F	27	visual loss	32*24*35	invasive	15	6	2+
2	M	28	headache	19*16*23	No	15	12	/
3	F	38	headache	21*17*14	No	15	12	3+
4	M	30	sexual dysfunction	28*16*21	No	15	24	1+
5	F	24	amenorrhea	27*17*13	No	15	10	0+
6	F	21	amenorrhea	24*11*38	No	15	28	2+
7	F	37	visual loss	30*34*40	invasive	15	4	3+
8	F	32	lactation	24*16*29	No	15	24	1+
9	F	29	amenorrhea	24*37*29	No	15	18	2+
10	M	31	sexual dysfunction	26*28*31	invasive	15	10	2+

Supplementary Table 2. List of primers for qPCR

species	Gene	sequences
rat	NEK2	F: ATGGCAGTTTGCAGGTGAGG<br>R: TGACATGGACGGAACCCTGT
rat	PTTG1	F: CTTGGGTCTCTCCCCTCAGT<br>R:GAGCCCAGCTTCAATCCATCC
rat	AURKA	F:GCCTTTCTGACCTGCTGGATG<br>R:TTGGCATGACATTTAGCCCTGG
rat	DNMT1	F:GACCAATGAGGCACTGTCCG<br>R:GACCGCGACTGCAATACACA
rat	ARID1B	F:GATCTCCCCACGGGAACAGA<br>R: GAGGTGGGGTGATGCATGTG

rat	GAPDH	F:CAAGTTCAACGGCACAGTCA<br>R:CCCCATTTGATGTTAGCGGG
rat	Axin2	F: ATGCCTATCAGGTGTTCTTG<br>R: CCACTCCTCTTCCTCATTCA
rat	CCND1	F: GGCGGATGAGAACAAGCAGA<br>R:ATGGAGGGTGGGTTGGAAAT
rat	c-myc	F:AAAGCCTAACCTCACAACCT<br>R:GAAAGAAGATGGGAAGCATA
human	NEK2	F: TGCTTCGTGAACTGAAACATCC<br>R: CCAGAGTCAACTGAGTCATCACT
human	β-actin	F: CATGTACGTTGCTATCCAGGC<br>R: CTCCTTAATGTCACGCACGAT
